# Supplementary material for: CD56 regulates human NK cell cytotoxicity through Pyk2
Source: eLife. 2020 Jun 8;9:e57346. doi: 10.7554/eLife.57346 (PMC7358009; doi:10.7554/eLife.57346)
Supplement: Supplementary file 1. [file elife-57346-supp1.pdf]

Key Resources Table				
**Reagent type (species) or resource**	**Designation**	**Source or reference**	**Identifiers**	**Additional information**
gene (*Homo sapiens*)	*NCAM1*	NCBI	NM_000615	
cell line (*Homo sapiens*)	NK92	American Type Culture Collection	Cat # CRL-2407; RRID:CVCL_2142	
cell line (*Homo sapiens*)	YTS	PMID:10403641	RRID:CVCL_D324	Dr. J. Orange (Columbia University); subline of YT originally derived in Dr. J. Strominger's lab
cell line (*Homo sapiens*)	NK92 CD56-KO	PMID:27435370		
cell line (*Homo sapiens*)	NK92 CD56-KO NCAM140-mApple	This paper	NM_000615	Dr. E. Mace (Columbia University); NK92 cell line with re-expression of full-length NCAM140
cell line (*Homo sapiens*)	NK92 CD56-KO NCAM140-mApple ΔECD	This paper		Dr. E. Mace (Columbia University); NK92 cell line with re-expression of NCAM140 with extracellular domain deleted

cell line (*Homo sapiens*)	NK92 CD56-KO NCAM140-mApple ΔICD	This paper		Dr. E. Mace (Columbia University); NK92 cell line with re-expression of NCAM140 with intracellular domain deleted
cell line (*Homo sapiens*)	YTS CD56-KO	This paper		Dr. E. Mace (Columbia University); YTS cell line with CD56 deleted
cell line (*Homo sapiens*)	721.221	PMID:3257565	RRID:CVCL_6263	Dr. J. Orange (Columbia University)
cell line (*Homo sapiens*)	K562	PMID:163658	RRID:CVCL_0004	Dr. J. Orange (Columbia University)
cell line (*Homo sapiens*)	KT86	PMID:17785506		Dr. J. Orange (Columbia University); K562 expressing CD86
cell line (*Homo sapiens*)	Phoenix	American Type Culture Collection	Cat #:CRL-3213; RRID:CVCL_H716	
cell line (*Homo sapiens*)	Raji	PMID:14304234	RRID:CVCL_0511	Dr. J. Orange (Columbia University)
cell line (*Homo sapiens*)	Jurkat	American Type Culture Collection	Clone E6-1; Cat#:TIB-152; RRID:CVCL_0367	
Biological sample (*Homo sapiens*)	Peripheral blood	Columbia University IRB AAAR7377; Baylor College of	Deidentified	

		Medicine IRB H30487		
Biological sample (*Homo sapiens*)	Leuko-reduction system (LRS) chambers	Mississippi Valley Regional Blood Center	Deidentified	
recombinant DNA reagent	U6gRNA-Cas9-2A-GFP (plasmid)	Sigma-Aldrich	Cat#: CAS9GFPP; Guide:CGCTGATCTCCCCCTGGCTGGG	CD56 targeting CRISPR plasmid
recombinant DNA reagent	CD56 (NCAM1) Human ORF clone	Origene	Cat#:RG213470; NM_000615, transcript variant 1	For subcloning to generate pBABE-puro-NCAM-mApple
recombinant DNA reagent	pBABE-puro-NCAM140 mApple (plasmid)	This paper		Produced by Epoch Life Sciences Inc. Available from Dr. E. Mace (Columbia University)
recombinant DNA reagent	pBABE-puro-NCAM140 ΔECD mApple (plasmid)	This paper		Produced by Epoch Life Sciences Inc. Available from Dr. E. Mace (Columbia University)
recombinant DNA reagent	pBABE-puro-NCAM140 ΔICD mApple (plasmid)	This paper		Produced by Epoch Life Sciences Inc. Available from Dr. E. Mace (Columbia University)
antibody	Anti-CD56 (mouse monoclonal; Alexa Fluor 647)	Biolegend	Clone HCD56; Cat# 318314, RRID:AB_604103	IF (1:100)
antibody	Anti-CD56 (mouse monoclonal; BV605)	Biolegend	Clone HCD56; Cat# 318334, RRID:AB_2561912	FC (1:200)

antibody	Anti-CD56 (mouse monoclonal; BV421)	Biolegend	Clone HCD56; Cat#:318328; RRID:AB_11218798	FC (1:100)
antibody	CD56 (mouse monoclonal; unconjugated)	Cell Signaling Technology	Clone:123C3; Cat#:3576S; RRID:AB_2149540	WB (1:1000)
antibody	Actin (rabbit polyclonal)	Sigma	Cat#:A2066 RRID:AB_476693	WB (1:4000)
antibody	RDye 680RD Goat anti-Mouse IgG	LiCOR Biosciences	925-68070; RRID:AB_2651128	WB (1:10,000)
antibody	IRDye 800CW Goat anti-Rabbit IgG	LiCOR Biosciences	Cat#:926-32211; RRID:AB_621843	WB (1:10,000)
antibody	Pyk2 phospho Y402 (rabbit polyclonal)	Abcam	Cat#:ab4800; RRID:AB_2173988	IF (1:100), FC (1:50)
antibody	PSA-NCAM (rat monoclonal)	BD Biosciences	Clone:12F8; Cat#:556325; RRID:AB_396363	FC (1:50)
antibody	Goat anti-Rat IgG (H+L) Secondary Antibody (FITC)	Thermo	Cat#:31629 RRID:AB_228240	FC (1:100)
antibody	PSA-NCAM (mouse monoclonal, unconjugated)	Millipore	Clone 2-2B; Cat#MAB5324; RRID:AB_95211	WB (1:1000)

antibody	CD18 (mouse monoclonal, unconjugated)	Produced from hybridoma; PMID:3594570	Clone:IB4; RRID:CVCL_0339	Cross-linking for activation on glass (10 µg/ml)
antibody	NKp30 (mouse monoclonal, unconjugated)	Biolegend	Clone:P30-15; Cat#:325202; RRID:AB_756106	Cross-linking for activation on glass (10 µg/ml)
antibody	Mouse IgG1aκ	Biolegend	Clone:MG1-45; Cat#:401402; RRID:AB_2801451	Isotype control for activation on glass (10 µg/ml)
antibody	CD55 (mouse monoclonal; PE)	Biolegend	Clone:JS11; Cat#311308; RRID:AB_314865	FC (1:100)
antibody	NKG2A (mouse monoclonal; Alexa Fluor 700	R&D Biosystems	Clone:131411; Cat# FAB1059N; RRID:AB_10972129	FC (1:50)
antibody	CD20 (mouse monoclonal; BV421)	Biolegend	Clone:2H7; Cat#:302330; RRID:AB_10965543	FC (1:50)
antibody	CD244 (mouse monoclonal; FITC)	BD Biosciences	Clone 2-69; Cat# 550815; RRID:AB_393900	FC (1:10)
antibody	CD158d (mouse monoclonal; APC)	R&D Biosystems	Clone 179315; Cat#:FAB1847A RRID:AB_2130821	FC (1:25)
antibody	CD122 (mouse monoclonal; PE)	Biolegend	Clone TU27; Cat #:339006; RRID:AB_2248892	FC (1:10)
antibody	CD94 (mouse monoclonal; APC)	Biolegend	Clone DX22; Cat#:305508 RRID:AB_2133129	FC (1:50)
antibody	CD45 (mouse monoclonal;	Beckman Coulter	Clone J.33; Cat#: B36294; RRID:AB_2833027	FC (1:20)

	Krome Orange)			
antibody	CD28 (mouse monoclonal; PE	BD Biosciences	Clone L293; Cat #:348047; RRID:AB_400368	FC (1:10)
antibody	CD18 (mouse monoclonal; APC)	BD Biosciences	Clone:6.7; Cat#:551060; RRID:AB_398485	FC (1:10)
antibody	CD11a (mouse monoclonal; PE Cy7)	BD Biosciences	Clone HI111; Cat#:561387; RRID:AB_2130821	FC (1:50)
antibody	CD2 (mouse monoclonal; Pacific Blue)	Beckman	Clone 39C1.5; Cat#:B09685; RRID:AB_2847880	FC (1:20)
antibody	Granzyme B (mouse monoclonal; PE Texas Red)	Thermo	Clone GB11; Cat#:GRB17 RRID:AB_1500187	FC (1:40)
antibody	Granzyme A (mouse monoclonal; Alexa Fluor 647)	Biolegend	Clone:CB9; Cat#:507214; RRID:AB_2114395	FC (1:40)
antibody	CD107a (LAMP-1) (mouse monoclonal; PE-Cy5)	Thermo	Clone:H4A3; Cat#:15-1079-42: RRID:AB_657557	FC (1:40)
antibody	Perforin (mouse monoclonal; FITC)	Biolegend	Clone:dG9; Cat#:308104; RRID:AB_314702	FC (1:12.5)
antibody	Interferon γ (mouse monoclonal; Alexa Fluor 700)	Biolegend	Clone:4S.B3; Cat#:502520; RRID:AB_528921	FC (1:33)
antibody	Perforin (mouse monoclonal; Alexa Fluor 488)	Biolegend	Clone:dG9; Cat#:308108; RRID:AB_493252	IF (1:50)

antibody	Tubulin (mouse monoclonal; biotin)	Thermo	Clone:236-10501; Cat#A21371; RRID:AB_2535843	IF (1:20)
antibody	Goat anti-rabbit IgG Alexa Fluor 488	Thermo	Cat#:A-11008; RRID:AB_143165	IF (1:100)
other	Streptavidin, Alexa Fluor 405 conjugated	Thermo	Cat#:S32351	IF (1:100)
other	Phalloidin, Alexa Fluor 568 conjugate	Thermo	Cat#:A12380	IF (1:100)
Peptide, recombinant protein	Recombinant human IL-15	Peprotech	Cat#:200-15	Cell culture (3-5 ng/ml)
Peptide, recombinant protein	Human IL-2	Sigma	Cat#:11147528001	^{51}Cr assays (1000 U/ml); NK92 maintenance (100 U/ml)
sequence-based reagent	CD56 gRNA	IDT	CGCUGAUCUCCCCCUGGCU	For primary cells
commercial assay or kit	IFNγ ELISA	Abcam	Cat#:ab46025	
chemical compound, drug	PF431396	Tocris	Cat#:4278	Pyk2 inhibitor
chemical compound, drug	Cr51	Perkin Elmer	Cat#: NEZ030S005MC	
chemical compound, drug	Peptide N-glycosylase (PNGase F)	New England Biolabs	Cat#:P0704S	FC (1 U/ml)
chemical compound, drug	Phospholipase C protein, phosphatidylinositol specific (PIPLC)	Thermo	Cat#:P6466	

software, algorithm	Fiji	PMID:22743772	RRID:SCR_002285	
software, algorithm	Imaris	Bitplane	RRID:SCR_007370	
software, algorithm	Image Studio Lite	Licor	RRID:SCR_013715	
software, algorithm	Prism 8.0	GraphPad Software	RRID:SCR_002798	
software, algorithm	Flowjo	BD Biosciences	RRID:SCR_008520	

FC: flow cytometry, WB: Western blotting, IF: immunofluorescence
